# Editorial: Patterning mechanisms in cardiovascular dysfunction: from bench to bedside

**DOI:** 10.3389/fcell.2026.2029645

**Published:** 2026-10-30

**Authors:** Virginia Actis Dato

**Affiliations:** School of Medicine, University of California San Diego, La Jolla, CA, United States

**Keywords:** cardiac function, cardiac tissue organization, cardiovascular disease, cardiovascular patterning, vascular patterning

## Introduction

The heart and blood vessels depend on intricate interactions between cells and molecular signals to develop, function and adapt to changing demands. Regulatory programs governing cardiovascular cell growth, organization and responses to stress are essential for preserving tissue architecture and function, whereas their disruption can contribute to heart failure, arrhythmias and atherosclerosis. Advances in developmental models, three-dimensional imaging, molecular approaches and *in vivo* cardiovascular phenotyping now provide increasingly detailed insight into the processes that shape cardiac development and vascular homeostasis. These approaches allow investigation of how cellular and molecular cues coordinate tissue formation, vascular function and remodeling, while revealing how altered regulation can drive cardiovascular dysfunction and disease progression. This Research Topic brought together studies examining how cellular, molecular and tissue-level processes influence cardiovascular development and disease, with an emphasis on developmental biology and preclinical disease models. Its themes included extracellular matrix remodeling during cardiac morphogenesis, regulatory processes underlying atherosclerosis, endothelial senescence and vascular remodeling, and pathways implicated in cardiovascular disease. The five contributions span embryonic cardiac matrix organization, vascular regulation, endothelial aging, transcriptional control of heart failure, and kinase-dependent regulation of cardiac function.

## From embryonic patterning to vascular gene regulation

Jorquera et al. characterized the second heart field (SHF)-associated dorsal pericardial wall (DPW) in *Xenopus laevis*, revealing stage-dependent changes in tissue architecture and extracellular matrix (ECM) organization. Between NF35 and NF42, ISL1^+^ SHF-associated cells transition from predominantly monolayered to multilayered arrangements, accompanied by changes in cell shape and polarity and by ECM remodeling within the DPW, with reduced fibronectin (Fn1) intercellular localization, increased intercellular tenascin-C (TnC), and increased collagen I levels at NF42. Global Fn1 depletion impaired cardiac morphogenesis, resulting in outflow tract shortening and reduced ventricular size and DPW area, together with decreased collagen I and, at the higher morpholino dose, decreased TnC levels. These findings support a role for Fn1 in ECM assembly and organization during SHF-associated morphogenesis, although the early, embryo-wide nature of the knockdown prevents attributing these effects specifically to the DPW. They also identify *Xenopus* as a useful comparative model for studying ECM-dependent cardiac development.

Casale et al. reviewed the multilayered regulation of LRP1 in atherosclerosis, highlighting its context-dependent roles in lipid metabolism, extracellular matrix turnover, and inflammatory signaling. The review identifies transcriptional regulation by metabolic and inflammatory cues, including SREBPs and HIF-1α, and non-coding RNAs as the best-supported regulatory layers, while also discussing post-translational mechanisms such as receptor shedding and degradation. Genetic variation provides additional evidence for enhancer-mediated regulation: the intronic variant rs11172113 promotes allele-specific recruitment of the transcriptional repressor SNAIL, reducing LRP1 expression and altering extracellular matrix remodeling programs in vascular smooth muscle cells. In contrast, direct evidence for DNA methylation and histone-mediated regulation at the LRP1 locus remains limited. The authors therefore highlight locus-specific epigenomic profiling, integrated single-cell multi-omics, and functional validation across vascular cell types as priorities for defining LRP1 regulatory architecture in atherosclerosis.

## Endothelial aging and a hypothesis for the vertebral artery

Wang et al. proposed a mechanobiological framework linking cervical spine degeneration, altered vertebral artery hemodynamics, and endothelial senescence. The review integrates evidence that chronic disturbed or oscillatory flow, mechanical stress, metabolic imbalance, and inflammation can promote endothelial dysfunction and senescence through pathways involving MEF2–KLF2/4, HDAC5/7, YAP/TAZ, and DNA damage responses such as p53/p21. The authors hypothesize that degeneration-associated changes in cervical anatomy and vertebral artery geometry may alter local shear stress and progressively remodel the vascular microenvironment, potentially compromising posterior circulation perfusion. However, this proposed link remains unproven, as direct evidence of endothelial senescence in human vertebral arteries associated with cervical degeneration is lacking. The framework therefore provides a hypothesis-generating basis for future mechanistic studies investigating the relationship between biomechanical remodeling, vascular aging, and cerebrovascular dysfunction.

## Transcriptional and kinase control of the failing and healthy heart

Wu et al. review the context-dependent role of c-Myc in heart failure, highlighting its involvement in hypertrophy, fibrosis, metabolic remodeling, inflammation, and cardiac repair. In HFrEF, increased c-Myc activity is associated with pathological remodeling and metabolic dysregulation, whereas other studies suggest protective effects in cardiac regeneration, which appears limited to specific conditions (moderate, transient expression, cooperation with other reprogramming factors, mainly neonatal hearts), and in doxorubicin-induced cardiomyopathy through reparative macrophages, although its role in cardiomyocytes remains controversial. Evidence in HFpEF remains limited and largely indirect, pointing to potentially divergent effects across metabolic, inflammatory, fibrotic, and endothelial pathways. Therapeutic strategies discussed include c-Myc-targeting siRNA, Omomyc, and compounds derived from traditional Chinese medicine; however, evidence in heart failure remains preclinical, and clinical data for Omomyc come from oncology. The review emphasizes that disease context and stage, cell type, expression level, and timing are critical determinants of c-Myc function, and that cell-type-specific modulation will be needed for translation.

Finally, Yarbro et al. investigated cardiac-specific Pak1 gain- and loss-of-function using transgenic and knockout mice. Pak1 overexpression produced mild concentric left ventricular remodeling, characterized by increased wall thickness and reduced chamber dimensions, without significant increases in myocardial fibrosis or ANP/BNP expression. Transgenic mice also exhibited higher fractional shortening, global longitudinal, radial and circumferential strain, +dP/dtmax, and several diastolic functional indices. Similar findings were observed in aged transgenic mice, although the cross-sectional design does not establish preservation with aging. Pak1 knockout mice showed modestly reduced systolic indices. The authors note that increased + dP/dtmax does not by itself establish load-independent intrinsic contractility.

## Conclusions and perspectives

Taken together, these five contributions illustrate how cardiovascular development and disease are shaped by interactions across multiple levels of biological regulation, encompassing extracellular matrix organization, transcriptional and post-transcriptional control, endothelial responses to mechanical stress, and kinase-dependent modulation of cardiac function. Three themes emerge. First, biological effects are strongly context dependent: the consequences of pathways involving c-Myc, LRP1, and Pak1 vary according to cell type, expression level, developmental or disease state, and timing. Second, extracellular matrix organization, cellular signaling, and tissue remodeling provide complementary perspectives for understanding cardiovascular development, dysfunction, and repair, although direct mechanistic links across these processes remain to be established. Third, distinguishing experimentally supported findings from proposed mechanisms is essential, particularly when evidence in the relevant tissue or disease context remains limited, as illustrated by the hypothesized relationship between cervical degeneration, vertebral artery hemodynamics, and endothelial senescence.

The breadth of this Research Topic reflects the value of integrating developmental models, molecular regulatory studies, mechanobiological frameworks, and *in vivo* cardiovascular phenotyping. Future studies should combine spatially and temporally restricted perturbations, locus-specific and single-cell multi-omic approaches, and direct validation of candidate pathways in disease-relevant tissues. Cell-type-specific manipulation of regulatory networks, together with rigorous assessment of cardiac and vascular function, will help determine which of these mechanisms represent actionable targets for cardiovascular disease.

